# An Advanced AIDS Patient With CD4 <20 and Plasmablastic Lymphoma Achieving Complete Response With the V-EPOCH Regimen

**DOI:** 10.7759/cureus.8641

**Published:** 2020-06-15

**Authors:** Blessie Nelson, Angelina Hong, Fatima Iqbal, Rohit Venkatesan

**Affiliations:** 1 Department of Hematology and Oncology, University of Texas Medical Branch at Galveston, Galveston, USA; 2 Department of Pathology and Laboratory Medicine, University of Texas Medical Branch at Galveston, Galveston, USA; 3 Department of Hematology and Oncology, University of Texas MD Anderson Cancer Center, Galveston, USA

**Keywords:** hiv aids, bortezomib, plasmablastic lymphoma, response, aids, cd4, v-epoch, chemotherapy

## Abstract

Plasmablastic lymphoma (PBL) is a rare form of non-Hodgkin lymphoma that is highly aggressive and carries a poor prognosis. Although the standard chemotherapy choice for most diffuse large B-cell lymphomas (DLBCL) is R-CHOP (rituximab, cyclophosphamide, doxorubicin, vincristine, and prednisone), subtypes of DLBCL such as PBL are less responsive to this treatment regimen. The preferred regimens for PBL include infusional EPOCH (etoposide, prednisone, vincristine, cyclophosphamide, and doxorubicin hydrochloride), HyperCVAD (cyclophosphamide, vincristine sulfate, doxorubicin hydrochloride, and dexamethasone), or CODOX-M/IVAC (cyclophosphamide, vincristine, doxorubicin, high‐dose methotrexate/ifosfamide, etoposide, and high‐dose cytarabine). Recent studies have begun to investigate the addition of other agents to these regimens to improve survival. This case report is about a patient with a history of advanced acquired immunodeficiency syndrome (AIDS) with a cluster of differentiation 4 (CD4) count <20 who had CD20 negative plasmablastic lymphoma and was successfully treated with the combination of bortezomib and dose-adjusted EPOCH (V-EPOCH) and intrathecal chemotherapy, achieving complete response with optimal tolerance. To our knowledge, this is the first case to demonstrate a complete response with V-EPOCH for PBL in advanced AIDS with CD4 <20. We aim to highlight the importance of standardizing effective chemotherapeutic approaches to this cancer entity and augment the effectiveness of V-EPOCH therapy in the literature review.

## Introduction

Plasmablastic lymphoma (PBL) is a rare form of non-Hodgkin lymphoma that is highly aggressive with no standardized regimens for treatment currently. Although the common chemotherapy choice for most diffuse large B-cell lymphomas (DLBCL) is R-CHOP (rituximab, cyclophosphamide, doxorubicin, vincristine, and prednisone), subtypes of DLBCL, such as PBL, are less responsive to CHOP (cyclophosphamide, doxorubicin hydrochloride, vincristine sulfate, and prednisone) therapy as per National Comprehensive Cancer Network (NCCN) guidelines. The preferred recommended regimens for PBL include infusional EPOCH (etoposide, prednisone, vincristine, cyclophosphamide, and doxorubicin hydrochloride), HyperCVAD (cyclophosphamide, vincristine sulfate, doxorubicin hydrochloride, and dexamethasone), or CODOX-M/IVAC (cyclophosphamide, vincristine, doxorubicin, high‐dose methotrexate/ifosfamide, etoposide, and high‐dose cytarabine) [1-3]. Recent studies have begun to investigate the addition of other agents to these regimens to improve survival. This case report will demonstrate the efficacy and tolerance of using bortezomib with EPOCH-based chemotherapy in the setting of advanced acquired immunodeficiency syndrome (AIDS) with cluster of differentiation 4 (CD4) count as low as below 20.

## Case presentation

**Figure 1:**
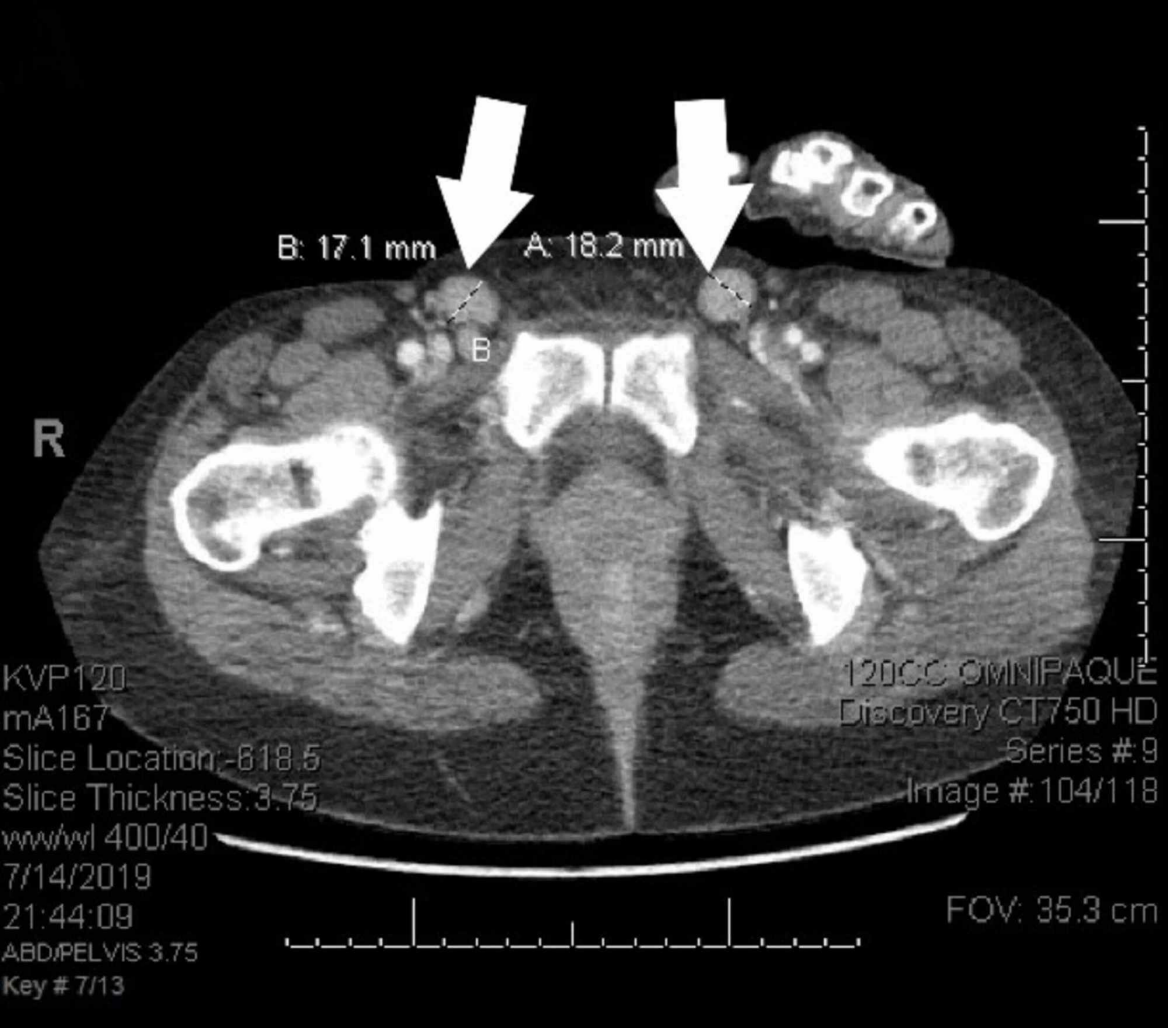
CT scans before treatment The patient’s initial CT scan showed bilateral inguinal lymphadenopathy with 18.2 mm (A) and 17.1 mm diameters (B) CT: computed tomography

**Figure 2:**
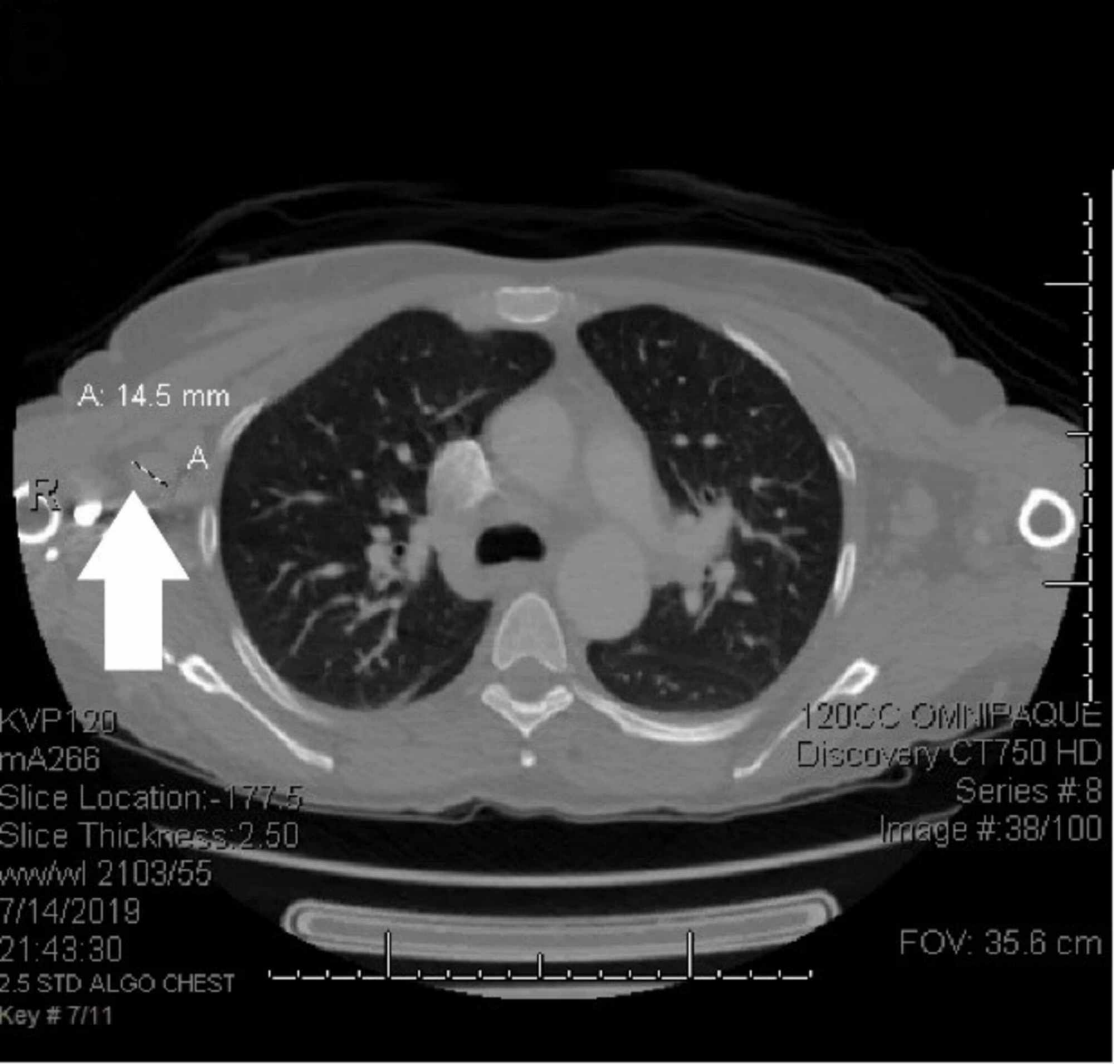
CT scans before treatment Scattered nodular lung opacities, hilar lymphadenopathy, and enlarged axillary nodes with the largest being 14.5 mm in diameter (A) CT: computed tomography

**Figure 3:**
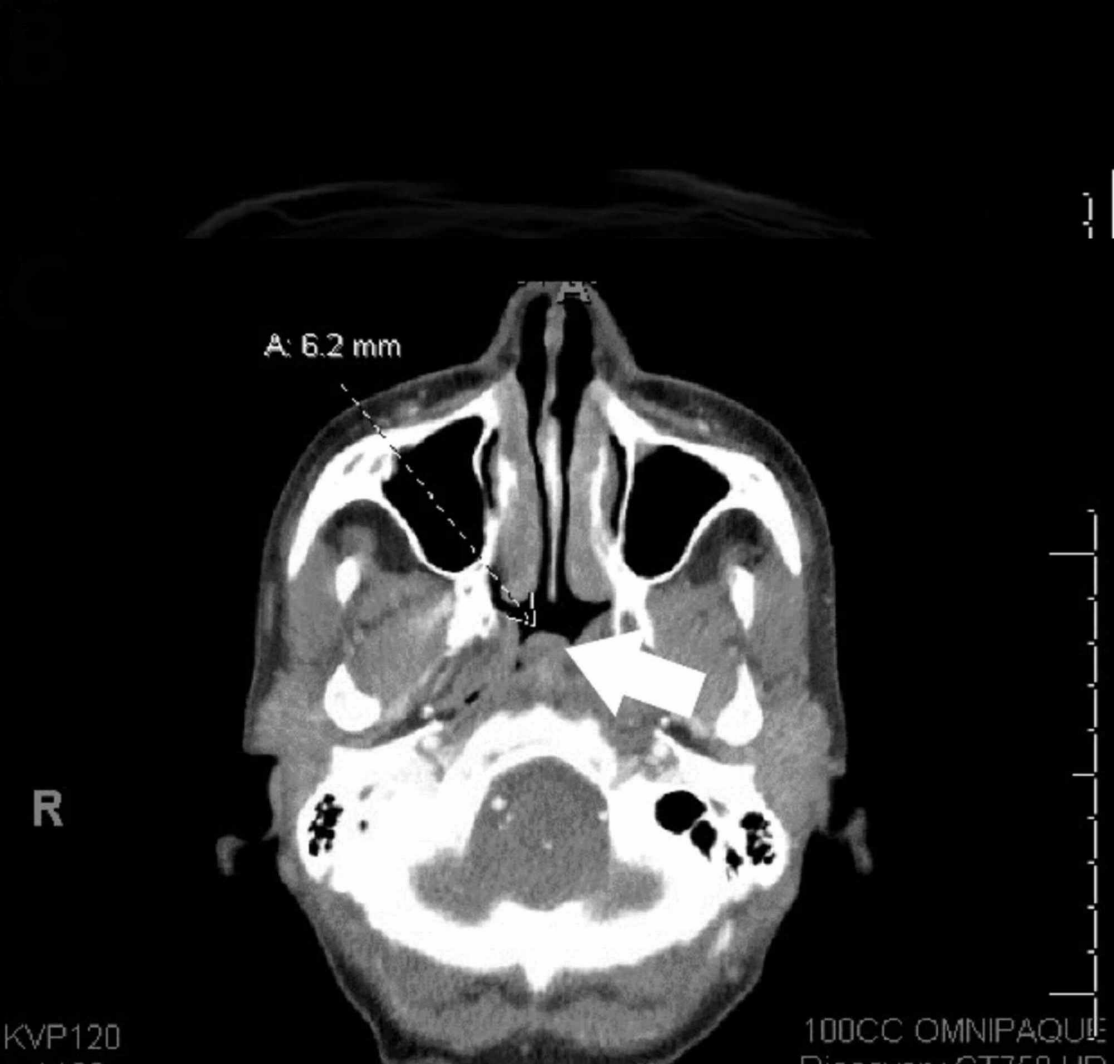
CT scans before treatment Heterogeneous cystic mass region in the nasopharynx (A) CT: computed tomography

**Figure 4:**
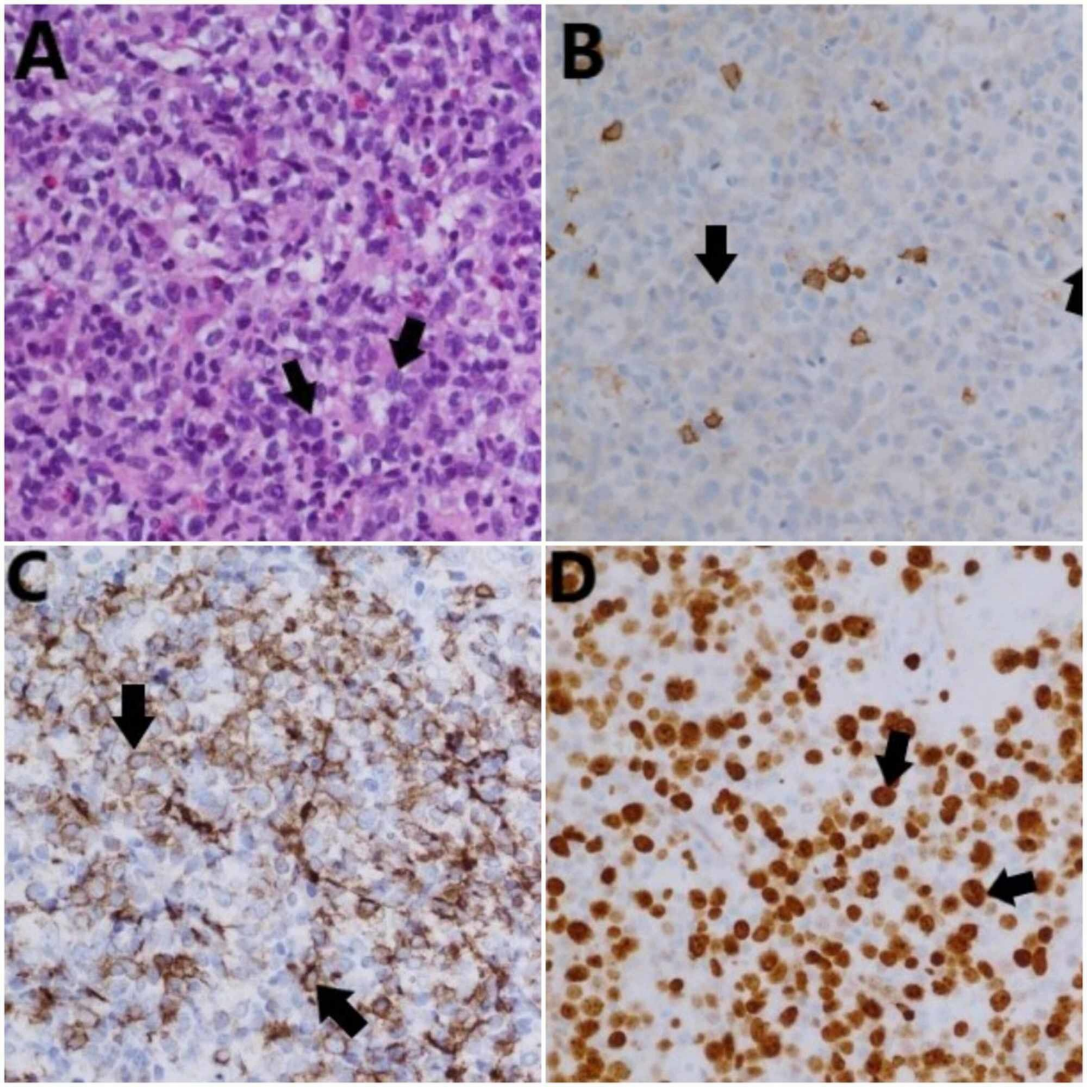
Pathology findings confirming plasmablastic lymphoma The patient’s right inguinal lymph node biopsy showed atypical lymphoid cells and admixed plasma cells (A), which were negative for CD20 (B), consistent with the characteristics of PBL. Low levels of CD138 were found in the atypical cells (C). Approximately 80%-90% of the abnormal lymphocytes had a positive Ki-67 stain (D). CD: cluster of differentiation; PBL: plasmablastic lymphoma

**Figure 5:**
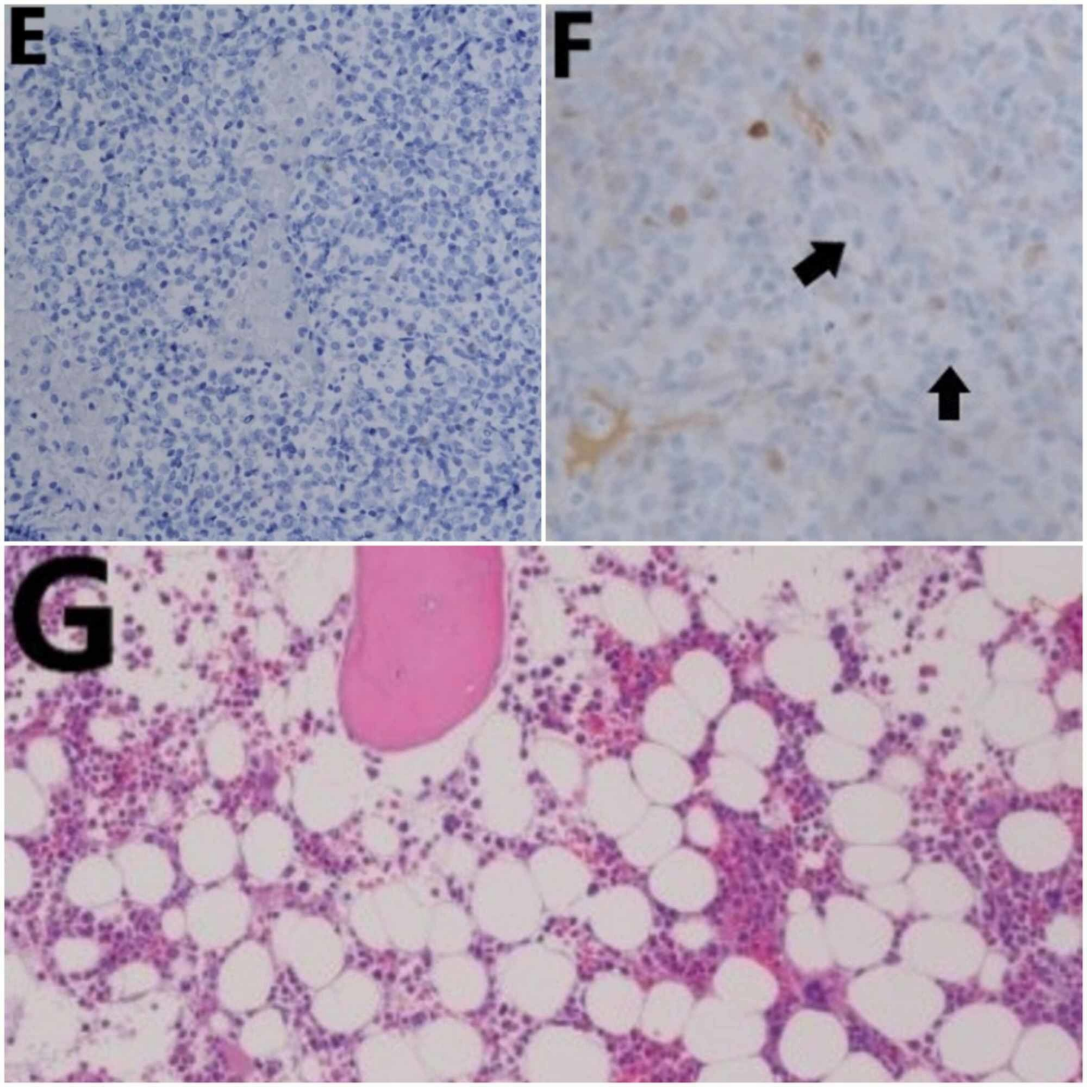
Pathology findings confirming PBL HHV-8 (E) and c-Myc (F) immunostaining were negative. Bone marrow biopsy, performed about seven days after she received her first cycle of chemotherapy, showed hypocellular marrow with trilineage hematopoiesis from myeloid cells (G). PBL: plasmablastic lymphoma; HHV-8: human herpesvirus 8

The patient was a 46-year-old African American female with a past medical history of AIDS, gastro-esophageal reflux disease (GERD), obstructive sleep apnea, and mild intermittent asthma who presented with diffuse lymphadenopathy and fever. She was diagnosed with human immunodeficiency virus (HIV) 20 years ago but was not compliant with her antiretroviral therapy (ART) for the past two years and restarted ART three weeks prior to presentation. On presentation, her HIV viral load was 61,918,000 copies/mL and CD4 count was 15 cells/uL (2%) (advanced AIDS). Computed tomography (CT) imaging of thorax, abdomen, and pelvis showed pulmonary nodules and retroperitoneal, iliac, inguinal, mediastinal, hilar, and axillary lymphadenopathy as seen in Figures 1-2, respectively. CT neck showed a right-sided cystic mass, as well as a heterogeneous cystic mass in the nasopharynx as seen in Figure 3. Her right inguinal lymph node biopsy was consistent with a high-grade B-cell lymphoma, most likely PBL, as seen in Figure 4A-4D, respectively. Lymphoplasmacytic infiltration was detected in the node with mild CD138 staining and CD20 negativity. Her bone marrow biopsy was negative for lymphoma involvement as seen in Figure 5E-5G, respectively. She was staged as a stage IIIB with an International Prognostic Index of 2 [4].

She was started on treatment with the V-EPOCH regimen with intrathecal chemotherapy. Her ART was changed from Genvoya® (Gilead Sciences, Inc., Foster City, California) to dolutegravir, lamivudine, and tenofovir to optimize chemotherapy for future cycles and avoid interactions with chemotherapy. Following Cycle 3, imaging findings were consistent with a near-complete response (CR); CT thorax showed that several of her pulmonary nodules had decreased in size and one had completely resolved. CT abdomen and pelvis showed resolution of retroperitoneal and pelvic adenopathy, and CT neck no longer showed the nasopharyngeal mass nor necrotic right lymph node. One month after Cycle 4, CD4 count was at 66 cells/uL and HIV viral load was undetectable. She went on to complete Cycles 5 and 6 with the V-EPOCH regimen. Repeat positron emission tomography (PET) after Cycle 6 showed a complete response with no PET-avid fluorodeoxyglucose (FDG) uptake anywhere.

## Discussion

The World Health Organization (WHO) defines PBL as a mature B-cell lymphoma (BCL) with plasma cell antigens that lacks certain B-cell antigens that are commonly found in other BCLs, such as CD19, CD20, and PAX5. PBL was originally described as residing in the oral cavity of HIV positive patients but is now known to also appear in other extranodal sites and in HIV negative patients [5-6]. Compared to more common forms of BCL and DLBCL, PBL has an aggressive course, with an overall survival rate of about six to 19 months. The NCCN now recommends infusional EPOCH, HyperCVAD, or CODOX-M/IVAC [1-3]. The most common treatment regimens for DLBCL and PBL are R-CHOP and CHOP, respectively; however, PBL is CD20 negative and usually does not respond to rituximab [7]. Intrathecal chemotherapy can also be used in conjunction with these regimens as prophylaxis against lymphoma affecting the central nervous system due to HIV status, which increases the central nervous system (CNS) risk [8]. Sparse published literature examining the impact of adding bortezomib to CHOP or EPOCH therapy in DLBCL patients has yielded mixed results [9-10]. A 2009 study demonstrated that R-CHOP with bortezomib resulted in an overall >75% CR rate in various types of B-cell lymphoma, with an 88% CR rate in patients with aggressive lymphoma such as DLBCL [9]. In contrast, in 2018, the first meta-analysis on the efficacy of bortezomib containing regimens in DLBCL patients undertaken concluded that adding bortezomib does not improve the overall survival rate in patients newly diagnosed with DLBCL or the ABC DLBCL subtype, which is the most common variation [10]. Bortezomib is classically used to treat multiple myeloma and mantle cell lymphoma. Because it is a proteasome inhibitor, bortezomib ultimately suppresses the NF-kB signaling pathway, leading to apoptosis of DLBCL cells that express NF-kB12 [11]. Furthermore, it is especially effective when combined with EPOCH because there is a synergistic relationship between bortezomib and the DNA-damaging effects of doxorubicin.

Despite the controversial efficacy of bortezomib in patients with DLBCL, recent literature has suggested that bortezomib is particularly beneficial for patients with PBL, significantly improving response and survival rates [12-13]. Bortezomib has shown clinical efficacy in other CD20-negative lymphomas, as well as post-germinal B-cell DLBCLs, further supporting its utility in treating PBL [14-16]. In 2016, Fedele et al. also proposed the use of V-EPOCH in PBL, with a case report describing an HIV-negative, 72-year-old male with stage IV PBL who was found to be in CR after six cycles of V-EPOCH and intrathecal methotrexate [13]. Castillo et al.’s 2015 case report first demonstrated the utility of bortezomib with EPOCH in PBL by describing three PBL patients (two who were HIV-positive with CD4 of 290 and 34, respectively, and one who was immunocompetent) who all achieved CR at 24, 18, and 12 months respectively [12]. In 2018, a retrospective analysis of 16 patients with PBL who received frontline V‐EPOCH was done with a median CD4 count of 128 with CR in 15 patients and partial response (PR) in one patient [17]. However, to our knowledge, this is the first case of PBL with advanced AIDS and CD4 counts <20, achieving complete response with V-EPOCH x six cycles.

## Conclusions

We present this case to illustrate how V-EPOCH can be safely used in an immunocompromised patient with advanced AIDS and a CD4 count <20 without significant adverse side effects and excellent efficacy. This patient’s complete response following bortezomib with dose-adjusted EPOCH therapy provides hope for patients with PBL and other rarer, more aggressive forms of DLBCL and encourages further long-term research exploring the side effects and long-term survival of V-EPOCH in PBL with severe immunocompromised states in establishing an acceptable and standardized regimen to improve survival rates and quality of life.
